# Role of serum EBV‐VCA IgG detection in assessing gastric cancer risk and prognosis in Northern Chinese population

**DOI:** 10.1002/cam4.1792

**Published:** 2018-10-10

**Authors:** Zeyang Wang, Zhi Lv, Hanxi Ding, Qian Xu, Liping Sun, Jingjing Jing, Yuan Yuan

**Affiliations:** ^1^ Tumor Etiology and Screening Department of Cancer Institute and General Surgery the First Hospital of China Medical University Shenyang China; ^2^ Liaoning Provincial Education Department Key Laboratory of Cancer Etiology and Prevention (China Medical University) Shenyang China; ^3^ Key Laboratory of Pathogen and Prevention of Digestive tract tumor in Liaoning Province Shenyang China

**Keywords:** gastric cancer, gastric function, *Helicobacter pylori*, prognosis, risk, serum EBV

## Abstract

The study aimed to investigate the role of serum EBV‐VCA IgG in assessing gastric cancer (GC) risk and prognosis. A total of 1790 Northern Chinese participants with pathologically confirmed disease underwent EBV‐VCA IgG serologic testing using enzyme‐linked immunosorbent assay (ELISA), including 821 controls, 410 atrophic gastritis (AG) patients, and 559 GC patients. We found that positive EBV‐VCA IgG was significantly associated with GC and its precursor, conferring a 1.55‐ and 1.36‐fold increased risk of GC and AG, respectively (*P* = 0.001, 95% CI = 1.21‐1.99; *P* = 0.011, 95% CI = 1.07‐1.72, respectively). The risk effects were more remarkable in younger, female, and *Helicobacter pylori*‐negative individuals than in older, male, and *H. pylori*‐positive individuals. EBV‐VCA IgG‐positive subjects had a lower PGI/II ratio than EBV‐VCA IgG‐negative subjects (median 8.0 vs 8.8, *P* = 0.001), especially those in the *H. pylori*‐positive (median 6.1 vs 6.8, *P* = 0.027) and GC subgroups (median 6.4 vs 7.9, *P* = 0.020). In the intestinal GC subgroup, the survival of EBV‐VCA IgG‐positive patients was worse than that of EBV‐VCA IgG‐negative patients (*P* = 0.041, HR = 2.45, 95% CI = 1.04‐5.78). Our study suggests that EBV‐VCA IgG seropositivity has potential in predicting the risk of GC and its precursor as well as the prognosis of histologically classified GC.

## Abstract 2

This is an innovative report about the role of serum EBV‐VCA IgG detection in assessing the risk of gastric cancer (GC) and its precursor as well as GC prognosis. And this is the first report about the relationship between EBV‐VCA IgG serology assay and gastric function status. Our study provides theoretical and experimental basis for evaluating the potential of serum EBV‐VCA IgG as a biomarker in prediction of GC risk and prognosis.

## 1 INTRODUCTION

Gastric cancer (GC) is the fifth most common cancer worldwide and the third leading cause of cancer‐related death.^1^, ^2^ As a complex disease, gastric carcinogenesis is characterized by a multistage process affected by multiple factors. Correa P has suggested that GC initiation and progression follow the cascade of superficial gastritis (SG)–atrophic gastritis (AG)–intestinal metaplasia (IM)–gastric dysplasia (GD)–GC.^3^ The susceptibility of individuals to GC is significantly elevated under precancerous conditions (AG, IM, and GD). It would thus be greatly beneficial for GC prevention and treatment to identify individuals at high risk of GC and block the progression of precancerous diseases.

The etiology of GC can be attributed to genetic, physical, chemical, and infective factors. Regarding infective factors, *Helicobacter pylori* (*H. pylori*), and Epstein‐Barr virus (EBV) have been well accepted as class I carcinogens. EBV belongs to the γ‐Herpes virus family, also called Herpesvirus 4, a human lymphocytic virus.^4^, ^5^ EBV infection is closely related to various types of human malignant tumors, such as Burkitt's lymphoma, nasopharyngeal cancer, and Hodgkin's and non‐Hodgkin's lymphoma.^6^, ^7^, ^8^, ^9^, ^10^ In 1993, EBV‐associated gastric carcinoma (EBVaGC) was first reported by Tokunaga.^11^, ^12^ In 2014, the Cancer Genome Atlas (TCGA) research group employed a variety of technologies to analyze the data of GC patients classified into four pathological subtypes of GC, among which the EBV‐associated GC subtype^13^ was paid great attention.

Serologic testing, a convenient, noninvasive, and cost‐effective technology, demonstrates significant advantages in population epidemiological surveys, dynamic monitoring of disease processes, and risk prediction. Thus far, the EBV serologic assay has been extensively applied to risk evaluation and early diagnosis of Burkitt lymphoma,^14^ nasopharyngeal cancer,^15^ Hodgkin's or non‐Hodgkin's lymphoma,^16^ and testicular cancer.^17^, ^18^ However, few studies have focused on the relationship between EBV serology and GC risk.^19^, ^20^, ^21^, ^22^ No investigation has referred to an association between either EBV serology and GC prognosis or EBV and serum gastric function indicators such as pepsinogen I (PGI), pepsinogen II (PGII), pepsinogen I/II (PGI/II), and gastrin‐17 (G‐17), which could reflect the functional status of gastric mucosa and were thus recognized as indicators for the early detection of GC.^23^ In addition, it remains unclear whether the EBV serologic assay has the potential to be a biomarker for GC prediction and prognosis.

Here, we conducted a population epidemiological survey and case–control study of Northern Chinese individuals to explore the association between EBV‐VCA IgG, which is an important component reflecting EBV infection, and prognosis as well as serum gastric function indicators, aiming to provide clues for the role of serum EBV‐VCA IgG in the prediction of GC risk and prognosis.

## 2 MATERIALS AND METHODS

### 2.1 Collection of the epidemiological and clinical information of participants

The study was approved by the Ethics Committee of the First Hospital of China Medical University. Informed consent was obtained from all participants. The epidemiological characteristics of the study subjects are presented in Table 1. A total of 1790 individuals were enrolled in our study, including 821 healthy controls, 410 AG patients, and 559 GC patients. For the control group, AG patients and a few GC patients were selected from The Zhuanghe GC Screening Program in Liaoning, China, between 1997 and 2016.^24^ The other GC cases were inpatients who underwent surgery at the First Hospital of China Medical University (Shenyang, Liaoning) during 1997‐2016. Epidemiological data and medical information for each subject were obtained from face‐to‐face inquiry or medical records. Individuals who smoked more than once a day and last >1 year were defined as ever smokers. And those who drank more than once a week and last >1 year were defined as ever drinkers. The others were grouped in never smokers or nondrinkers, respectively. Fasting venous blood samples were collected from each subject. Gastroscopy and biopsy were conducted for all patients, and histopathological examination was carried out by two independent pathologists. The control group was confirmed to have normal stomach or mild superficial gastritis. AG was diagnosed according to the Sydney classification,^25^, ^26^ and participants with moderate to severe AG were selected as the AG group. Based on postoperative pathological examination, GC was classified according to the World Health Organization criteria^27^ and Lauren's classification^28^ and was further divided into intestinal‐type and diffuse‐type GC. Tumor staging was determined according to the 7th edition of the TNM staging system of the International Union Against Cancer (UICC)/American Joint Committee on Cancer (AJCC) (2010).^29^ Follow‐up was completed by 30 June 2017. Finally, 234 patients with available information were included in the survival analysis.

**Table 1:** The baseline of the studied patients

Variables	CON (%)	AG (%)	GC (%)
Sex			
Male	419 (51.0)	256 (62.4)	373 (66.7)
Female	402 (49.0)	154 (37.6)	186 (33.3)
Age			
Mean ± SD	52.5 ± 10.1	52.9 ± 9.8	58.3 ± 10.8
Age range	16‐85	30‐77	21‐87
*Helicobacter pylori*			
Positive	222 (27.0)	227 (55.4)	306 (54.7)
Negative	599 (73.0)	183 (44.6)	253 (45.3)
Smoking			
Never	281 (68.9)	1 (100.0)	145 (66.5)
Ever	127 (31.1)	0 (0.0)	73 (33.5)
Drinking			
Never	319 (78.2)	1 (100.0)	160 (73.7)
Ever	89 (21.8)	0 (0.0)	57 (26.3)

AG, atrophic gastritis; CON, control group, normal stomach mucosa; GC, gastric cancer.

### 2.2 Detection of EBV‐VCA IgG and PGI, PGII, G17, and Hp‐IgG in serum using ELISA

We detected EBV‐VCA immunoglobulin G (EBV‐VCA IgG) antibody titer by enzyme‐linked immunosorbent assay (ELISA, Origene, Rockville, MD, USA). The procedure was performed according to the manual, including a negative control, positive control, and calibrator sample. The OD was read at 450 nm using an ELISA reader (Multiskan Ascents 354, Thermo LabSystems, Waltham, MA USA) within 15 minutes. OD >1.1 was considered EBV‐positive based on the instructions.^19^, ^30^, ^31^, ^32^, ^33^ Aside from differences in the incubation time and temperature, we also tested the serum PGI, PGII, G17, and *H. pylori* immunoglobulin G (*H. pylori*‐IgG) antibody titers by ELISA (PGI kit; PGII kit; G17 kit; Helicobacter pylori‐IgG kit, Biohit, Helsinki, Finland) according to previously described reports.^23^ *H. pylori*‐IgG titer >34 IU was diagnosed as *H. pylori* positive according to the manufacturer's reagent specification sheet.

### 2.3 Quality control of ELISA

Quality control of the ELISA was carried out during the entire test process. Regarding the quality of tested samples, EBV‐VCA IgG seropositivity was compared by time period (1997‐2000, 2001‐2005, 2006‐2010, and 2011‐2016), and no difference was found between the different time periods (Table S1). With respect to the sensitivity of the ELISA kit, we randomly selected a 10% subset from all tested samples and retested them with a different kit (Cusabio, Wuhan, China). The consistency of these two tests was 95.7% (Table S2). Duplicate negative and positive controls were included in each 96‐well test plate. Valid results met the following criteria: the OD of the calibrator was >0.25; that of the negative control was <0.9; and that of the positive control was >1.2. The samples that yielded implausible values were retested.

### 2.4 Statistical analysis

Tests of normality were performed for serum EBV‐VCA IgG titer, *H. pylori*‐IgG titer, PGI, PGII, and G17. When a quantitative comparison was performed, the median with the 25% quartile and 75% quartile was adopted due to the non‐normality of these indicators. A nonparametric Mann‐Whitney *U* test was employed to evaluate the difference between two groups. Unconditional logistic regression was used to calculate ORs and 95% confidence intervals (CIs) for prediagnostic EBV serostatus and GC risk adjusted by age, gender, and *H. pylori* status. The relationship between EBV‐VCA IgG and gastric function was explored using correlation analysis. A Kaplan‐Meier test was used to examine the association of EBV‐VCA IgG with the overall survival of GC patients. Cox regression was applied to perform multivariate analysis while controlling for the clinicopathological parameters related to prognosis. All statistical analyses were performed using PASW Statistics for Windows, version 18.0 (SPSS Inc, Chicago, IL, USA). All tests were two‐sided, and statistical significance was set as *P* < 0.05.

## 3 RESULTS

### 3.1 Baseline characteristics of the subjects

The data of subjects for risk analysis are shown in Table 1, including age, gender, and *H. pylori* infection, smoking and drinking status for the control, AG and GC groups. The data of subjects for the analysis of prognosis are shown in Table S3, including clinicopathological parameters such as macroscopic type, Lauren classification, TNM stage, growth pattern, depth of invasion, lymphatic metastasis, and lymphovascular invasion. Among these parameters, macroscopic type, TNM stage, depth of invasion, and lymphatic metastasis were associated with overall survival and were regarded as adjustment factors when investigating EBV infection and prognosis.

### 3.2 Distribution characteristics of serum EBV‐VCA IgG in Northern Chinese Individuals

First, we analyzed the distribution of serum EBV‐VCA IgG based on age, gender, and smoking and drinking status in Northern Chinese individuals, including qualitative analysis (EBV‐VCA IgG was categorized as positive or negative according to the cut‐off value on the kit) and quantitative analysis (based on EBV‐VCA IgG titer). The results suggested that the EBV‐VCA IgG‐positive rate of the older group (>60 years) was significantly higher than that of the younger group (39.1% vs 33.5%, *P* = 0.021). A similar phenomenon was also observed in EBV‐VCA IgG titer (55.6 vs 48.2, *P* = 0.023). No significant difference was found in the qualitative and quantitative results of the EBV‐VCA IgG assay based on gender, smoking, and drinking status (*P* > 0.05, Table 2).

**Table 2:** The baseline characteristics on qualitative and quantitative of serum EBV‐VCA IgG test

	N (%)	EBV ‐VCA IgG quantitative	*P* a	EBV ‐VCA IgG qualitative		*P* b	OR (95% CI)
		Median (25% quartiles, 75% quartiles)		Negative	Positive		
Total	1790 (100.0)	50.8 (21.2, 94.4)		1160 (64.8)	630 (35.2)		
Age (yr)							
≦60	1243 (69.4)	48.2 (20.4, 93.0)	**0.023**	827 (66.5)	416 (33.5)	**0.021**	
>60	547 (30.6)	55.6 (24.4, 96.9)		333 (60.9)	214 (39.1)		
Gender							
Male	1048 (58.5)	50.0 (20.5, 91.3)	0.321	690 (65.8)	358 (34.2)	0.276	
Female	742 (41.5)	51.1 (22.0, 99.1)		470 (63.3)	272 (36.7)		
*Helicobacter pylori*							
Seropositive	755 (42.2)	52.1 (23.2, 93.8)	0.368	478 (63.3)	277 (36.7)	0.259	
Seronegative	1035 (57.8)	49.5 (19.9, 95.0)		682 (65.9)	353 (34.1)		
Smoking							
Never smoker	427 (68.1)	47.0 (19.9, 88.1)	0.230	299 (69.5)	128 (65.0)	0.255	
Ever smoker	200 (31.9)	54.2 (25.8, 91.7)		131 (30.5)	69 (35.0)		
Drinking							
Nondrinker	480 (76.7)	50.1 (22.1, 88.4)	0.442	331 (77.2)	149 (75.6)	0.676	
Drinker	146 (23.3)	43.7 (19.2, 88.4)		98 (22.8)	48 (24.4)		
Disease				158 (64.2)	88 (35.8)		
CON	821 (42.2)	49.0 (19.8, 93.4)	1 (ref.)	571 (69.5)	250 (30.5)		1 (ref.)
AG	410 (21.1)	50.8 (22.7, 98.4)	0.200	246 (60.0)	164 (40.0)	**0.001**	**1.55 (1.21‐1.99)**
GC	559 (28.7)	52.1 (23.3, 93.6)	0.155	343 (61.4)	216 (38.6)	**0.011**	**1.36 (1.07‐1.72)**
Intestinal‐type GC	246 (58.4）	58.8 (29.6, 104.2)	**0.019**	71 (55.5)	57 (44.5)	**0.015**	**1.63 (1.10‐2.42)**
Diffuse‐type GC	128 (30.4)	48.3 (20.0, 86.9)	0.864	158 (64.2)	88 (35.8)	0.257	1.20 (0.88‐1.63)
Mix‐type GC	47 (11.2)	35.0 (9.0, 74.9)	0.106	35 (74.5)	12 (25.5)	0.304	0.70 (0.35‐1.39)
GC vs CON+AG			0.558			0.147	1.17 (0.94‐1.46)
GC+AG vs CON			0.101			**<0.001**	**1.45 (1.18‐1.77)**

AG, atrophic gastritis; CON, control group, normal stomach mucosa; GC, gastric cancer.

a *P*‐values to compare the quantitative of EBV between different ages, gender, *H. pylori* infection status, smoking, drinking, and between normal stomach mucosae (CON) and other gastric diseases by the Mann‐Whitney U test. Values are medians (with 25%–75% quartiles). When quantitative comparisons are made, medians as well as the 25% quartile and the 75% quartile were used because of the non‐normality of these indicators.

b *P*‐values to compare EBV positive/negative between different ages, gender, *H. pylori* infection status, smoking, drinking, by the χ2 test, and between normal stomach mucosae (CON) and other gastric diseases by the multifactorial logistic regression adjusted by age and sex.

The bold font means the significant results.

### 3.3 Association of serum EBV‐VCA IgG with GC and AG risk

We found that the positive rate of EBV‐VCA IgG was significantly higher in both the AG and GC groups than in the control group (AG: 40.0% vs 30.5%; GC: 38.6% vs 30.5%). After adjustments for age and gender, EBV‐VCA IgG seropositivity could elevate the risk of AG, GC, and especially intestinal‐type GC by 1.55‐, 1.36‐, and 1.63‐fold, respectively (*P* = 0.001, 95% CI = 1.21‐1.99; *P* = 0.011, 95% CI = 1.07‐1.72; *P* = 0.015, 95% CI = 1.10‐2.43, respectively, Table 2). Stratified analysis was further performed based on age, gender, and smoking, drinking and *H. pylori* infection status. It was suggested that the contribution of EBV‐VCA IgG to AG and GC risk was statistically significant in the young, female, and *H. pylori*‐negative subgroups but not in the smoking and drinking subgroups (*P* > 0.05). In the young subgroup, EBV‐VCA IgG seropositivity conferred 1.53‐ and 1.62‐fold increased AG and GC risk, respectively (*P* = 0.004, 95% CI = 1.14‐2.04; *P* = 0.001, 95% CI = 1.21‐2.17, respectively), and the EBV‐VCA IgG titer in GC patients was significantly higher than that in the control group (median 53.3 vs 46.8, *P* = 0.031). The subjects with EBV‐VCA IgG seropositivity had 1.90‐ and 1.52‐fold increased risks of AG and GC in the female subgroup (*P* = 0.001, 95% CI = 1.30‐2.79; *P* = 0.029, 95% CI = 1.04‐2.21, respectively) compared to those negative for EBV‐VCA IgG, and the risks of AG and GC were increased by 1.61‐ and 1.41‐fold in the *H. pylori‐*negative subgroup, respectively (*P* = 0.007, 95% CI = 1.14‐2.27; *P* = 0.036, 95% CI = 1.02‐1.95, respectively, Table 3).

**Table 3:** The stratified cerum EBV‐VCA IgG test

Disease	N (%)	EBV‐VCA IgG quantitative	*P* a	EBV ‐VCA IgG qualitative		*P* b	OR (95% CI)
				Negative	Positive		
Stratified by age							
≦60							
CON	629 (50.6)	46.8 (18.6, 88.7)	1 (ref.)	449 (71.4)	180 (28.6)		1 (ref.)
AG	306 (24.6)	48.3 (22.3, 97.8)	0.159	190 (62.1)	116 (37.9)	**0.004**	**1.53 (1.14‐2.04)**
GC	308 (24.8)	53.3 (23.7, 96.9)	**0.031**	188 (61.0)	120 (39.0)	**0.001**	**1.62 (1.21‐2.17)**
GC vs CON+AG			0.161			**0.015**	**1.40 (1.01‐1.83)**
GC+AG vs CON			**0.029**			**<0.001**	**1.58 (1.24‐2.01)**
>60							
CON	192 (35.1)	59.0 (27.6, 100.2)	1 (ref.)	122 (63.5)	70 (36.5)		1 (ref.)
AG	104 (19.0)	55.0 (23.5, 98.8)	0.941	56 (53.8)	48 (46.2)	0.054	1.63 (0.99‐2.67)
GC	251 (45.9)	51.8 (21.2, 91.5)	0.236	155 (61.8)	96 (38.2)	0.640	1.10 (0.74‐1.63)
GC vs CON+AG			0.128			0.792	0.95 (0.68‐1.35)
GC+AG vs CON			0.385			0.255	1.24 (0.86‐1.79)
Stratified by gender							
Male							
CON	419 (40.0)	48.6 (18.0, 91.7)	1 (ref.)	293 (69.9)	126 (30.1)		1 (ref.)
AG	256 (24.4)	43.8 (21.0, 92.5)	0.941	162 (63.3)	94 (36.7)	0.085	1.34 (0.96‐1.86)
GC	373 (35.6)	54.2 (26.0, 88.5)	0.236	235 (63.0)	138 (37.0)	0.135	1.26 (0.93‐1.71)
GC vs CON+AG			0.165			0.357	1.14 (0.87‐1.49)
GC+AG vs CON			0.385			0.059	1.30 (0.99‐1.69)
Female							
CON	402 (54.2)	50.3 (22.0, 97.1)	1 (ref.)	278 (69.2)	124 (30.8)		1 (ref.)
AG	154 (20.8)	56.2 (25.6, 108.7)	0.159	84 (54.5)	70 (45.5)	**0.001**	**1.90 (1.30‐2.79)**
GC	186 (25.0)	47.4 (19.0, 101.9)	**0.031**	108 (58.1)	78 (41.9)	**0.029**	**1.52 (1.04‐2.21)**
GC vs CON+AG			0.077			0.245	1.23 (0.87‐1.75)
GC+AG vs CON			0.029			**0.001**	**1.67 (1.23‐2.26)**
Stratified by smoking							
Never Smoker							
CON	281 (65.8)	46.7 (22.0,92.6)	1 (ref.)	201 (71.5)	80 (28.5)		1 (ref.)
GC	145 (34.0)	41.9 (19.1,83.0)	0.390	97 (66.9)	48 (33.1)	0.328	1.25 (0.80‐1.96)
GC vs CON+AG						0.323	1.25 (0.80‐1.97)
GC+AG vs CON						0.359	1.23 (0.79‐1.93)
Ever Smoker							
CON	127 (63.5)	58.1 (25.1,93.4)	1 (ref.)	84 (66.1)	43 (33.9)		1 (ref.)
GC	73 (36.5)	49.5 (26.0,87.0)	0.482	26 (35.6)	47 (64.4)	0.933	0.97 (0.52‐1.84)
Stratified by drinking							
Nondrinker							
CON	319 (66.5)	51.4 (23.1,87.8)	1 (ref.)	227 (71.2)	92 (28.8)		1 (ref.)
GC	160 (33.3)	48.9 (20.2,87.8)	0.614	103 (64.4)	57 (35.6)	0.168	1.34 (0.88‐2.04)
GC vs CON+AG						0.165	1.35 (0.89‐2.05)
GC+AG vs CON						0.187	1.33 (0.87‐2.01)
Drinker							
CON	89 (61.0)	55.4 (23.4,76.2)	1 (ref.)	58 (65.2)	31 (34.8)		1 (ref.)
GC	57 (39.0)	36.6 (23.4,76.2)	0.273	40 (70.2)	17 (29.8)	0.381	0.72 (0.34‐1.51)
Stratified by *H. pylori*							
*H. pylori* （−）							
CON	599 (57.9)	48.1 (18.6, 94.1)	1 (ref.)	132 (71.0)	54 (29.0)		1 (ref.)
AG	183 (17.7)	49.0 (22.3, 98.5)	0.382	54 (58.7)	38 (41.3)	**0.007**	**1.61 (1.14‐2.27)**
GC	253 (24.4)	51.8 (24.8, 96.0)	0.142	52 (59.8)	35 (40.2)	**0.036**	**1.41 (1.02‐1.95)**
GC vs CON+AG			0.198			0.111	1.28 (0.95‐1.74)
GC+AG vs CON			0.132			**0.002**	**1.51 (1.15‐1.97)**
*H. pylori* （+）							
CON	222 (29.4)	52.1 (24.7, 90.6)	1 (ref.)	153 (68.9)	69 (31.1)		1 (ref.)
AG	227 (30.1)	51.8 (22.9, 98.4)	0.587	137 (60.4)	90 (39.6)	0.061	1.46 (0.98‐2.16)
GC	306 (40.5)	53.5 (22.2, 91.6)	0.791	188 (61.4)	118 (38.6)	0.184	1.30 (0.88‐1.90)
GC vs CON+AG			0.912			0.784	1.05 (0.76‐1.43)
GC+AG vs CON			0.658			0.075	1.36 (0.97‐1.92)

AG, atrophic gastritis; CON, control group, normal stomach mucosae; GC, gastric cancer.

a *P*‐values to compare the quantitative of EBV between different diseases when stratified by ages, sexes, and statuses of *H. pylori* infection by the Mann‐Whitney U test. Values are medians (with 25%‐75% quartiles).

b *P*‐values to compare EBV positive/negative between different diseases when stratified by ages, sexes, and statuses of *H. pylori* infection by the χ^2^ test.

The bold font means the significant results.

### 3.4 Association of serum EBV‐VCA IgG with serum gastric function indicators PGI, PGII, PGI/II, and G17

To explore the relationship between serum EBV‐VCA IgG and gastric function indicators, we analyzed the expression levels of serum PGI, PGII, PGI/II, and G17 based on EBV‐VCA IgG status. The serum PGI/II ratio was significantly decreased in EBV‐VCA IgG‐positive subjects compared with EBV‐VCA IgG‐negative subjects (median 8.0 vs 8.8, *P* = 0.001), while no significant difference was observed in PGI, PGII, and G17 (*P* > 0.05). Stratified analysis was further performed based on *H. pylori* infection status and gastric diseases. The serum PGI/II ratio was significantly decreased in EBV‐VCA IgG‐positive subjects compared with EBV‐VCA IgG‐negative subjects both in the *H. pylori*‐positive (median 6.1 vs 6.8, *P* = 0.027) and GC subgroups (median 6.4 vs 7.9, *P* = 0.020, Table 4).

**Table 4:** The expression of gastric function indicates in different EBV‐VCA IgG carriers

Group	PGI		PGII		PGI/II		G17	
	Median (25%, 75%)	*P*	Median (25%, 75%)	*P*	Median (25%, 75%)	*P*	Median (25%, 75%)	*P*
Total	84.5 (60.1, 116.5)		9.3 (5.8, 16.6)		8.7 (5.4, 13.3)		4.0 (1.1, 18.4)	
EBV‐VCA								
EBV‐VCA (−)	87.1 (62.5, 119.8)	0.112	9.8 (5.9, 16.5)	0.218	8.8 (5.6, 13.4)	**0.001**	3.8 (0.9, 17.4)	0.992
EBV‐VCA (+)	83.4 (59.1, 115.9)		9.8 (5.9, 17.8)		8.0 (5.0, 12.1)		3.7 (1.1, 15.7)	
Stratified by *H. pylori*								
*H. pylori* (−)								
EBV‐VCA (−)	81.5 (60.0, 114.5)	0.213	7.5 (5.1, 11.9)	0.785	10.6 (7.0, 15.2)	0.115	3.5 (0.8, 11.7)	0.864
EBV‐VCA (+)	79.4 (54.0, 110.3)		7.7 (5.2, 13.1)		10.0 (6.3, 13.9)		3.4 (1.1, 11.7)	
*H. pylori* (+)								
EBV‐VCA (−)	92.4 (64.8, 125.4)	0.438	13.6 (8.0, 20.6)	0.207	6.8 (4.6, 10.4)	**0.027**	4.5 (1.1, 51.7)	0.358
EBV‐VCA (+)	88.2 (60.3, 119.1)		14.3 (8.4, 23.0)		6.1 (4.3, 9.5)		3.8 (1.0, 41.9)	
Stratified by Diseases								
CON								
EBV‐VCA (−)	83.9 (64.2, 111.7)	0.919	14.2 (9.3, 20.8)	0.945	11.3 (7.5, 16.0)	0.884	5.3 (1.1, 21.4)	0.433
EBV‐VCA (+)	84.2 (60.9, 112.7)		14.5 (8.9, 21.0)		10.9 (8.1, 15.0)		5.7 (1.4, 19.9)	
AG								
EBV‐VCA (−)	81.7 (62.6, 113.1)	0.064	13.7 (9.0, 21.4)	0.914	6.1 (4.3, 8.7)	0.298	1.6 (0.0, 3.4)	0.599
EBV‐VCA (+)	91.4 (66.3, 122.6)		16.8 (8.1, 22.1)		5.6 (4.1, 8.6)		1.3 (0.0, 3.3)	
GC								
EBV‐VCA (−)	85.2 (44.8, 135.3)	0.385	11.4 (5.7, 19.7)	0.669	7.9 (4.9, 12.3)	**0.020**	8.7 (2.7, 36.6)	0.546
EBV‐VCA (+)	77.7 (41.9, 118.3)		11.5 (5.7, 21.7)		6.4 (4.1, 10.5)		9.6 (2.6, 43.8)	

*P*‐values to compare the quantitative of gastric panel when stratified by EBV‐VCA status, or statuses of *H. pylori* infection and gastric diseases by the Mann‐Whitney U test.

The bold font means the significant results.

### 3.5 Association of serum EBV‐VCA IgG with serum H. pylori‐IgG

To explore the interactions among GC‐related infective factors, the relationship between serum EBV‐VCA IgG and *H. pylori*‐IgG was further analyzed. Without considering diseases, we investigated the distribution of serum EBV‐VCA IgG between *H. pylori*‐IgG‐positive and *H. pylori*‐IgG‐negative groups. No difference in serum EBV‐VCA IgG was observed between the groups (*P* = 0.259, Table 2 **)**. Correlation analysis also demonstrated no statistical significance, regardless of whether the overall population or the subgroups were analyzed (*P* > 0.05, Table S4). However, in the *H. pylori*‐negative subgroup, EBV‐VCA IgG conferred 1.61‐ and 1.41‐fold increased risks of AG and GC (*P* = 0.007, *P* = 0.036, respectively, Table 3), respectively; in the *H. pylori‐*positive subgroup, the serum PGI/II ratio in the positive EBV‐VCA IgG subgroup was significantly decreased compared with that in the negative EBV‐VCA IgG subgroup (6.1 vs 6.8, *P* = 0.027, Table 4).

### 3.6 Association of serum EBV‐VCA IgG with GC clinicopathological parameters

To explore the association between serum EBV‐VCA IgG and GC clinicopathological parameters, GC patients were grouped by tumor location, macroscopic type, Lauren classification, TNM stage, growth pattern, depth of invasion, lymphatic metastasis, lymphovascular invasion, etc The results showed that serum EBV‐VCA IgG seropositivity was associated with the depth of invasion (*P* < 0.001). The number of EBV‐VCA IgG‐positive patients in the pT1+pT2 subgroup was much greater than that of those in the pT3+pT4 subgroup (47.4% vs 31.8%). However, no association was found between serum EBV‐VCA IgG seropositivity and the other clinicopathological parameters (*P* > 0.05, Table 5).

**Table 5:** Associations between EBV‐VCA status and clinicopathological parameters of gastric cancer

Parameters	EBV‐VCA			*P*‐value
	Negative	Positive		
Location				
Body	29 (59.2)	20 (40.8)	0.271	
Angle	19 (63.3)	11 (36.7)		
Antrum	66 (73.3)	24 (26.7)		
Entire	6 (54.5)	5 (45.5)		
Macroscopic type				
Early stage	21 (75.0)	7 (25.0)		0.053
Borrmann I	2 (66.7)	1 (33.3)		
Borrmann II	34 (52.3)	31 (47.7)		
Borrmann III	80 (65.6)	42 (34.4)		
Borrmann IV	14 (87.5)	2 (12.5)		
TNM stage				
I	30 (61.2)	19 (38.8)		0.944
II	43 (65.2)	23 (34.8)		
III	65 (65.0)	35 (35.0)		
IV	13 (68.4)	6 (31.6)		
I_II	73 (63.5)	42 (36.5)		0.741
III+IV	78 (65.5)	41 (34.5)		
Growth pattern				
Massive	3 (60.0)	2 (40.0)		0.763
Nested	24 (64.9)	13 (35.1)		
Diffused	89 (70.1)	38 (29.9)		
Depth of invasion				
Mucous and submucosal layer	21 (75.0)	7 (25.0)		**<0.001**
Muscular layer	9 (31.0)	20 (69.0)		
Subserosa layer	22 (59.5)	15 (40.5)		
Serosal layer or invasion adjacent organs	98 (70.5)	41 (29.5)		
Mucous, submucosal and muscular layer (pT1+ pT2)	30 (52.6)	27 (47.4)		**0.033**
Subserosa, serosa or invasion adjacent organs (pT3+pT4)	120 (68.2)	56 (31.8)		
Lymphatic metastasis				
Negative	61 (63.5)	35 (36.5)		0.792
Positive	90 (65.2)	48 (34.8)		
Lymphovascular invasion				
Negative	79 (69.3)	35 (30.7)		0.879
Positive	23 (67.6)	11 (32.4)		
Smoking				
Never Smoker	97 (66.9)	48 (33.1)		0.712
Ever Smoker	47 (64.4)	26 (35.6)		
Drinking				
Nondrinker	88 (62.4)	53 (37.6)		0.536
Drinker	33 (67.3)	16 (32.8)		
Family history^a^				
No	107 (69.5)	47 (30.5)		0.684
Yes	13 (65.0)	7 (35.0)		

a When the immediate family of the patients died because of cancer, these patients was defined as family history positive.

The bold font means the significant results.

### 3.7 Association of serum EBV‐VCA IgG with GC prognosis

Follow‐up was conducted for 234 GC patients with complete clinicopathological data and information on death or survival. The follow‐up ended on 30 June 2017, and ranged from 6 to 204 months (the mean month was 48, and the median month was 39). First, we performed Kaplan‐Meier regression analysis for clinicopathological parameters and overall survival of GC. After adjustments for parameters affecting overall survival, univariate and multivariate analyses were performed for serum EBV‐VCA IgG status and GC prognosis. It was suggested that EBV‐VCA IgG seropositivity was not associated with overall survival. In stratified analysis, however, we found that the survival of EBV‐VCA IgG‐positive subjects was worse than that of EBV‐VCA IgG‐negative subjects in the intestinal GC subgroup (mean survival month: 48.0 vs 51.0, *P* = 0.041, HR = 2.45, 95% CI = 1.04‐5.78, Table 6).

**Table 6:** Univariate and multivariate cox proportional hazard analysis for EBV‐VCA infection status and GC overall survival

Variables	All GC	Death	MST^a^	Univariate		Multivariate^c^	
	n (%)	n	(M)	*P‐*value	Hazard ratio (95% CI)	*P‐*value	Hazard ratio (95% CI)
EBV‐VCA	n = 234	n = 90					
Negative	151 (64.5)	58	52	0.966	1 (Ref)	0.233	1 (Ref)
Positive	83 (35.5)	32	51.8^b^		1.01 (0.66‐1.55)		1.32 (0.84‐2.06)
Stratified analysis							
Macroscopic type							
Borrmann 0	n = 28	n = 1					
EBV‐VCA (−)	21 (75)	0	NA	0.608	1 (Ref)	NA	1 (Ref)
EBV‐VCA (+)	7 (25)	1	NA		NA		NA
Borrmann I+II	n = 68	n = 22					
EBV‐VCA (−)	36 (52.9)	11	63.6^b^	0.851	1 (Ref)	0.852	1 (Ref)
EBV‐VCA (+)	32 (47.1)	11	57.6^b^		1.08 (0.47‐2.50)		1.09 (0.45‐2.60)
Borrmann Ⅲ+Ⅳ	n = 138	n = 67					
EBV‐VCA (−)	94 (68.1)	47	38.0	0.988	1 (Ref)	0.372	1 (Ref)
EBV‐VCA (+)	44 (31.9)	20	36.0		1.00 (0.59‐1.68)		1.28 (0.74‐2.21)
Lauren classification							
Intestinal	n = 64	n = 24					
EBV‐VCA (−)	40 (62.5)	14	48.0	0.480	1 (Ref)	**0.041**	1 (Ref)
EBV‐VCA (+)	24 (37.5)	10	51.0^b^		0.75 (0.33‐1.68)		**2.45 (1.04‐5.78)**
Diffuse	n = 150	n = 62					
EBV‐VCA (−)	98 (65.3)	42	50.0	0.408	1 (Ref)	0.817	1 (Ref)
EBV‐VCA (+)	52 (34.7)	20	45.3^b^		1.25 (0.73‐2.14)		0.94 (0.55‐1.61)
Mixed	n = 20	n = 4					
EBV‐VCA (−)	13 (65.0)	2	74.5^b^	0.433		0.210	1 (Ref)
EBV‐VCA (+)	7 (35.0)	2	42.0^b^		0.46 (0.06‐3.25)		6.34 (0.35‐113.50)
TNM stage							
I+II	n = 115	n = 23					
EBV‐VCA (−)	73 (63.5)	15	65.8^b^	0.96	1 (Ref)	0.475	1 (Ref)
EBV‐VCA (+)	42 (36.5)	8	67.3^b^		1.02 (0.43‐2.41)		1.38 (0.57‐3.35)
III+IV	n = 119	n = 67					
EBV‐VCA (−)	78 (65.5)	43	24.0	0.977	1 (Ref)	0.577	1 (Ref)
EBV‐VCA (+)	41 (34.5)	24	24.0		0.99 (0.60‐1.64)		1.17 (0.68‐2.00)
Growth pattern							
Massive+Nested	n = 42	n = 13					
EBV‐VCA (−)	27 (64.3)	10	47.0	0.197	1 (Ref)	0.860	1 (Ref)
EBV‐VCA (+)	15 (35.7)	3	48.0^b^		0.43 (0.12‐1.56)		1.16 (0.22‐6.13)
Diffused	n = 127	n = 51					
EBV‐VCA (−)	89 (70.1)	37	50.0	0.823	1 (Ref)	0.346	1 (Ref)
EBV‐VCA (+)	38 (29.9)	14	53.0		1.07 (0.58‐1.99)		1.36 (0.72‐2.56)
Depth of invasion							
pT1+pT2	n = 94	n = 15					
EBV‐VCA (−)	52 (55.3)	7	67.7^b^	0.447	1 (Ref)	0.999	1 (Ref)
EBV‐VCA (+)	42 (44.7)	8	76.7^b^		1.48 (0.54‐4.09)		1.00 (0.14‐7.19)
pT3+pT4	n = 139	n = 75					
EBV‐VCA (−)	98 (70.5)	51	29.0	0.274	1 (Ref)	0.471	1 (Ref)
EBV‐VCA (+)	41 (29.5)	24	24.0		1.31 (0.81‐2.14)		1.19 (0.74‐1.91)
Lymphatic metastasis							
Negative	n = 96	n = 21					
EBV‐VCA (−)	61 (63.5)	13	64.6^b^	0.607	1 (Ref)	0.601	1 (Ref)
EBV‐VCA (+)	35 (36.5)	8	47.3^b^		1.26 (0.52‐3.05)		1.29 (0.49‐3.38)
Positive	n = 138	n = 69					
EBV‐VCA (−)	90 (65.2)	45	32.0	0.745	1 (Ref)	0.292	1 (Ref)
EBV‐VCA (+)	48 (34.8)	24	32.0		0.92 (0.56‐1.51)		1.33 (0.78‐2.25)
Lymphovascular invasion							
Negative	n = 113	n = 38					
EBV‐VCA (−)	78 (69.0)	26	52.0	0.542	1 (Ref)	0.274	1 (Ref)
EBV‐VCA (+)	35 (31.0)	12	53.0		1.24 (0.62‐2.46)		1.49 (0.73‐3.03)
Positive	n = 34	n = 11					
EBV‐VCA (−)	23 (67.6)	8	20.0	0.438	1 (Ref)	0.344	1 (Ref)
EBV‐VCA (+)	11 (32.4)	3	35.0^b^		0.59 (0.16‐2.24)		0.43 (0.08‐2.45)

CI, confidence interval; HR, hazard rate; NA, not available.

a MST, median survival time (months).

b Mean survival time was provided when MST could not be calculated.

c Multivariate survival analysis was carried out by adding the EBV‐VCA variable to the clinicopathological parameters with *P* < 0.05.

The bold font means the significant results.

## 4 DISCUSSION

Based on a large‐scale epidemiological survey and case–control study, we conducted a systematic investigation of the association of serum EBV‐VCA IgG status with the prediction of GC risk and prognosis. In addition, we primitively explored the relationship between serum EBV‐VCA IgG and gastric function indicators, including PGI, PGII, PGI/II, and G17, as well as EBV‐VCA IgG *and H. pylori*‐IgG.

### 4.1 Association of serum EBV‐VCA IgG with GC and AG risk

Epstein‐Barr virus contains various common antigens, such as viral capsid antigen (VCA), EBV nuclear antigen (EBNA), early antigen (EA), and latent membrane protein 1 (LMP1). To date, a few reports have focused on the relationship between the serologic EBV antibody test and GC risk. In 1995, Levine et al^19^ first detected serum VCA‐IgA, EA and EBNA in 54 GC subjects and 54 controls and found that the EBV antibody titer in GC subjects was significantly higher than that in age‐ and gender‐matched controls. Second, in 2007, American scholars detected VCA‐IgA, EA‐D IgA, VCA IgG, EA‐D IgG, EA‐R IgG, and EBNA in 185 GC subjects and 200 controls from an area in China where GC is common, and no significance difference was observed between participant groups.^20^ Third, in 2009, Korean scholars detected VCA IgG, VCA‐IgA, EBNA‐IgG, and EA‐IgG in 100 GC subjects and 200 controls from a cohort study involving 14440 subjects, and no associations were found between those parameters and GC risk.^21^ Fourth, in 2016, the latest report from Africa suggested that the positive rate of EBNA‐1 and EA was associated with GC risk in 51 GC subjects and 96 controls of the Zambia population in Africa.^22^ In our study, serum EBV‐VCA IgG was detected in 1790 pathologically confirmed Northern Chinese patients with GC and precancerous diseases using ELISA. The results showed that the positive rates of EBV‐VCA IgG in both the AG and GC groups were higher than those in the control group, and a similar trend was also demonstrated in the results of the EBV‐VCA IgG titer test. Positive EBV‐VCA IgG could increase AG and GC risk by 1.55‐ and 1.36‐fold, respectively; positive EBV‐VCA IgG especially increased intestinal‐type GC risk by 1.63‐fold. Stratified analysis based on age and gender further indicated that EBV‐VCA IgG‐infected subjects had higher GC risk in the younger subgroup than the older subgroup and that both the risks of AG and GC were increased in the female subgroup. It has been reported that primary EBV infection mainly occurs in childhood and reaches a summit before 20 years due to low immunity following infection.^34^ Then, the severity of infection decreases, with no emerging infection occurring over 60 years of age. Therefore, younger subjects are more likely than older subjects to be infected by EBV, but EBV is more likely to be detected in older subjects than younger subjects due to accumulating effects. Existing studies have reported that the EBV titer increases in male and old subgroups.^35^, ^36^, ^37^, ^38^ Generally, the factors without distinguished effects are more likely than factors with distinguished effects to manifest in the subgroup of background effects after removal of the risk of accumulative factors. Therefore, the high‐risk effects of EBV became more distinct in female and younger subgroups after removal of the male and older subgroups from our study. Further study is required to explain the conflict demonstrated in the association of EBV infection with age and gender.

### 4.2 Association of serum EBV‐VCA IgG with serum gastric function indicators

Pepsinogen (PG) and gastrin (G17) are effective indicators that reflect gastric function. In the present study, we first explored whether EBV infection could lead to changes in gastric function status. Serum PGI, PGII, PGI/II, and G17 were tested using ELISA. All subjects were categorized as EBV‐VCA IgG positive and EBV‐VCA IgG negative. We found that the serum PGI/II ratio in EBV‐VCA IgG‐positive subjects was decreased, while other indicators showed no significant difference. Stratified analysis based on disease classification further suggested that in the GC subgroup, the serum PGI/II ratio in the EBV‐VCA IgG‐positive group was lower than that in the EBV‐VCA IgG‐negative group. The PGI/II ratio is a more powerful indicator of gastric function. Reduction in the PGI/II ratio demonstrated a strong association with the genesis and development of GC.^39^ In the present study, we found preliminary evidence that serum EBV‐VCA IgG is correlated with a low PGI/II ratio. Further investigations are needed to explore the specific mechanism.

### 4.3 Association of serum EBV‐VCA IgG with serum H. Pylori‐IgG

*H. pylori* is the most important environmental factor for GC and is known as a type I carcinogenic factor. EBV is also considered to be involved in gastric carcinogenesis. To date, many studies have focused on *H. pylori* and EBV as well as the coinfection of both the pathogens, but the contribution of their coinfection to GC development remains unclear. Minoura‐Etoh et al^40^ reported that the products of *H. pylori* infection could activate EBV latency in gastric mucosal epithelial cells. Levine et al^19^ reported in a cohort study that individuals with a high titer of *H. pylori*‐IgG developed EBV‐negative GC rather than EBV‐positive GC. Camargo et al^41^ examined the association between *H. pylori* serologic antibody and EBV tissue in situ with 15 types of *H. pylori* serologic antibody, and no significant association was found. Other studies have also suggested that *H. pylori* infection is not associated with EBV infection.^21^, ^22^, ^42^, ^43^ However, recent studies have revealed that coinfection of EBV and *H. pylori* increased GC incidence and reduced the age at GC detection compared with individual infection.^44^ In our study, no significant association was observed between the seropositivity of EBV‐VCA IgG and *H. pylori*‐IgG in the overall population regardless of disease factors. No difference was found in the EBV‐VCA IgG titer between the *H. pylori*‐IgG‐positive and *H. pylori*‐IgG‐negative groups (Table 2), and correlation analysis of these factors also showed no statistically significant results (Table S4). Thus, we have not been able to draw a definite conclusion about whether a relationship exists between *H. pylori* and EBV infection on the basis of the current study due to the comparison of only two serum antibodies. Moreover, we found that EBV‐positive subjects had a lower serum PGI/II ratio in the *H. pylori*‐positive subgroup (including GC and AG) than in the *H. pylori‐*negative subgroup, indicating that *H. pylori* might synergistically influence gastric mucosa function, which could provide research clues for the interaction between EBV and *H. pylori*. More rigorous study designs and experiments are warranted to confirm the association between coinfection of EBV and *H. pylori* and GC development in the future.

### 4.4 Association of serum EBV‐VCA IgG with GC prognosis

The relationship between EBV infection and GC prognosis remains unclear. A meta‐analysis has demonstrated that EBV infection has protective effects on GC prognosis (overall survival).^45^ Camargo et al^46^ conducted histological EBV detection and correlation analysis with GC prognosis for 4599 GC patients worldwide and found that EBV‐positive GC subjects exhibited better survival than EBV‐negative GC subjects. Koriyama et al^47^ noted that EBV‐positive subjects with diffusive GC exhibited better survival than those with intestinal GC by means of EBV tissue in situ. To date, no investigation related to the relationship between the EBV serologic test and GC prognosis has been reported. Compared with histopathological markers, serologic assays are a convenient, noninvasive, and rapid early‐monitoring method that can dynamically evaluate the survival status of cancer patients preoperatively and postoperatively. In the present study, we systematically analyzed the relationship between serum EBV‐VCA IgG titer and overall survival in GC subjects. It was suggested that EBV‐VCA IgG seropositivity is not associated with overall survival. However, in the intestinal GC subgroup, EBV‐VCA IgG‐positive patients exhibited worse survival than EBV‐VCA IgG‐negative patients, which was consistent with the histological in situ findings reported by Koriyama C. Intestinal GC and diffusive GC have different origins, which may account for the different mechanism of their interactions with EBV. From this perspective, the serologic assay of EBV may become a potential biomarker for the prediction of GC prognosis in some histology‐based subgroups.

Some limitations in this study should be acknowledged. First, other than VCA IgG, multiple specific antibodies for EBV antigens, such as VCA‐IgA, EBNA, EA, and LMP, can be used in serological tests to define the EBV infection status. Our study focused only on VCA IgG, aiming to evaluate the application value of serum EBV‐VCA IgG as a marker instead of comprehensively assessing EBV infection and the target disease. Second, our study investigated whether VCA IgG played a prognostic role based only on the serological assay, a convenient, noninvasive, and rapid early‐monitoring method. In situ hybridization (ISH) of tissue to determine EBV‐associated gastric cancer (EBVaGC), which is a gold benchmark in the determination of EBV infection, is lacking in this study. Third, the present study was mainly concerned about whether the serum EBV‐VCA IgG could distinguish GC from AG and whether it is related to the serum gastric function indicators and clinical pathological parameters of GC. We did not identify EBVaGC cases among all the GC patients involved in the study using more accurate detection methods, such as EBV DNA detection. Thus, in‐depth studies are warranted in the future.

Finally, it must be noted that the EBV‐VCA IgG detection rate is relatively low (35% positive rate) in our study. EBV is a ubiquitous human herpes DNA virus that establishes a lifelong, persistent infection in over 90% of the population worldwide.^48^, ^49^ The most plausible explanation for the low detection rate may be the low quality of the samples or the low sensitivity of the serologic test. To interpret the logical reasoning, we checked for EBV‐VCA IgG seropositivity from two aspects. With respect to the quality of tested samples, we analyzed EBV‐VCA IgG seropositivity according to time period (1997‐2000, 2001‐2005, 2006‐2010, and 2011‐2016), and no difference was found between different time periods in the whole population (Table S1). Regarding the sensitivity of the ELISA test, a 10% subset was randomly selected from all tested samples and retested with a different kit (Cusabio, Wuhan, China). The results showed that the consistency of these two tests was 95.7% (Table S2). Furthermore, through a literature review, we found that the positive rate of EBV‐VCA worldwide varied based on ethnicity and country and was relatively high in Thailand but low in America.^36^, ^50^ In Thailand, the positive rate of EBV‐VCA IgG was more than 90% in childhood, reaching 100% in adults over 40 years of age. A study of EBV‐VCA IgG detection in Minnesota, USA, showed that the positive rate of EBV‐VCA IgG was 74% in non‐Hispanic Blacks, 62% in Asians, 50% in Spanish individuals, and 26% in non‐Hispanic Whites.^36^ Although the two verification experiments mentioned above reported similar results as previous studies and provided supportive evidence that the EBV detection rate in the region was low, a uniform error still could not be eliminated in the present study.

## 5 CONCLUSION

In summary, we examined serum EBV‐VCA IgG in 1790 Northern Chinese individuals following pathological confirmation of the dynamic disease chain control‐AG (precancerous disease)‐GC. We also explored the relationship of serum EBV‐VCA IgG with serum gastric function indicators, clinicopathological parameters, and the prognosis of GC patients. The results showed that positive EBV‐VCA IgG was associated with an increased risk of GC and its precursor AG, which was more notable in younger, female, and *H. pylori*‐negative individuals than in older, male, and *H. pylori‐*positive individuals. EBV‐VCA IgG‐positive subjects demonstrated a lower serum PGI/II ratio, which was more distinct in *H. pylori*‐positive GC patients than in *H. pylori‐*negative GC patients. In the intestinal GC subgroup, EBV‐VCA IgG‐positive subjects exhibited worse survival than EBV‐VCA IgG‐negative subjects. Our study provides a theoretical and experimental basis for evaluating the potential of serum EBV‐VCA IgG as a biomarker in predicting GC risk and prognosis. However, more rigorous experiments are necessary to verify our findings in the future.

## CONFLICT OF INTERESTS

The authors have declared that no competing interests exist.
