# Supplementary material for: Role of serum EBV‐VCA IgG detection in assessing gastric cancer risk and prognosis in Northern Chinese population
Source: Cancer Med. 2018 Oct 10;7(11):5760–74. doi: 10.1002/cam4.1792 (PMC6246934; doi:10.1002/cam4.1792)
Supplement: Supplementary file 1 [file CAM4-7-5760-s001.docx]

| Supplementary Table 1. The seropositivity of EBV in different time periods | | | | |
| --- | --- | --- | --- | --- |
|  | Years | | | |
| EBV | 1997-2000 | 2001-2005 | 2006-2010 | 2011-2016 |
|  | *P* = 0.829 | | | |
| Positive (%) | 212 (34.7) | 139 (33.7) | 163 (35.4) | 169 (36.7) |
| Negative (%) | 399 (65.3) | 273(66.3) | 298 (64.6) | 292 (63.3) |
|  |  |  |  |  |
| Stratified by disease |  |  |  |  |
| GC | *P* = 0.167 | | | |
| Positive (%) | 20 (43.5) | 23 (39.0) | 16 (57.1) | 157 (36.9) |
| Negative (%) | 26 (56.5) | 36 (61.0) | 12 (42.9) | 269 (63.1) |
| AG | *P* = 0.219 | | | |
| Positive (%) | 104 (37.0) | 34 (49.3) | 23 (41.8) | 3 (60.0) |
| Negative (%) | 177 (63.0) | 35 (50.7) | 32 (58.2) | 2 (40.0) |
| CON | *P* = 0.382 | | | |
| Positive (%) | 61 (28.1) | 74 (28.4) | 112 (33.8) | 3 (25.0) |
| Negative (%) | 156 (71.9) | 187 (71.6) | 219 (66.2) | 9 (75.0) |
| GC, gastric cancer; AG, atrophic gastritis; CON, control group, normal stomach mucosa. | | | | |

| Supplementary Table 2. The comparison of EBV-VCA IgG ELISA test using different ELISA kit | | | | |
| --- | --- | --- | --- | --- |
|  |  | EBV kit (Cat No. EA100941) | |  |
|  |  | Positive | Negative | Total |
| ELISA kit (Cat No. CSB-E05010h) | Positive | 74 | 7 | 81 |
|  | Negative | 1 | 104 | 105 |
|  | Total | 75 | 111 | 186 |
| Note: 186 patients sera were tested by ELISA kit, Origene, Rockville, USA (Cat No. EA100941) and another ELISA kit, Wuhan, China (Cat No. CSB-E05010h). 74 sera were positive and 104 were negative by both methods. The agreement is 95.7%. | | | | |

| Supplementary Table 3. Gastric cancer patient clinical features and univariate analysis of overall survival | | | | |
| --- | --- | --- | --- | --- |
|  | All GC | Death | MST^a^ |  |
| Factors | n=234(%) | n=90 | (M) | *P-value*^c^ |
| Macroscopic type |  |  |  |  |
| Early Stage | 28(12.0) | 1 | 56.2^b^ | **6.5×10^-5^** |
| Borrmann Ⅰ | 3(1.3) | 1 | 20.0 |  |
| Borrmann Ⅱ | 65(27.8) | 21 | 63.1^b^ |  |
| Borrmann Ⅲ | 122(52.1) | 59 | 36.0 |  |
| Borrmann Ⅳ | 16(6.8) | 8 | 38.0 |  |
| Lauren grade |  |  |  |  |
| Intestinal | 69(29.5) | 25 | 57.9^b^ | 0.407 |
| Diffuse | 165(70.5) | 65 | 53.0 |  |
| TNM stage |  |  |  |  |
| Ⅰ | 49(20.9) | 2 | 76.8^b^ | **1.6×10^-7^** |
| Ⅱ | 66(28.2) | 21 | 58.6^b^ |  |
| Ⅲ | 100(42.8) | 54 | 26.0 |  |
| Ⅳ | 19(8.1) | 13 | 15.0 |  |
| Ⅰ+Ⅱ  Ⅲ+Ⅳ |  |  |  | 6.1×10^-10^ |
|  |  |  |  |  |
| Growth pattern |  |  |  |  |
| Massive | 5(2.1) | 3 | 26.0 | 0.130 |
| Nested | 34(14.5) | 10 | 45.4^b^ |  |
| Diffused | 127(54.4) | 51 | 50.0 |  |
| Depth of invasion |  |  |  |  |
| Mucous and submucosal layer (pT1) | 28(12.0) | 1 | 56.2^b^ | **1.2×10^-7^** |
| Muscular and subserosa layer (pT2) | 66(28.3) | 14 | 71.6^b^ |  |
| Serosal layer or invasion adjacent organs (pT3+pT4) | 139(59.7) | 75 | 28.0 |  |
| pT1+pT2 vs. pT3+pT4 |  |  |  | 2.4×10^-9^ |
| Lymphatic metastasis |  |  |  |  |
| Negative | 96(41.0) | 21 | 64.2^b^ | **7.6×10^-6^** |
| Positive | 138(59.0) | 69 | 32.0 |  |
| Lymphovascular invasion |  |  |  |  |
| Negative | 113(76.9) | 38 | 53.0 | 0.139 |
| Positive | 34(14.5) | 11 | 38.0 |  |
| ^a^,MST, median survival time (months).^b^, mean survival time was provided when MST could not be calculated. ^c^, The *P* value was a estimation which comprehensive consideration the two factors for Death and MST. | | | | |

| Supplementary Table 4. The association of EBV-VCA IgG and gastric function indicates | | | | | |
| --- | --- | --- | --- | --- | --- |
|  | EBV -VCA IgG quantitative | |  | EBV -VCA IgG qualitative | |
|  | *P* | r |  | *P* | r |
| Total |  |  |  |  |  |
| PGI | 0.190 | -0.032 |  | 0.310 | 0.025 |
| PGII | 0.773 | 0.007 |  | 0.411 | -0.020 |
| PGI/II | 0.287 | -0.026 |  | 0.163 | 0.034 |
| G17 | 0.509 | -0.016 |  | 0.533 | 0.015 |
| HP +/- |  |  |  | 0.362 | 0.022 |
| CON |  |  |  |  |  |
| PGI | 0.598 | 0.018 |  | 0.712 | -0.013 |
| PGII | 0.853 | 0.007 |  | 0.772 | -0.010 |
| PGI/II | 0.709 | -0.013 |  | 0.705 | 0.013 |
| G17 | 0.319 | -0.050 |  | 0.559 | -0.021 |
| HP +/- |  |  |  | 0.814 | -0.008 |
| AG |  |  |  |  |  |
| PGI | 0.385 | -0.043 |  | 0.172 | 0.068 |
| PGII | 0.522 | -0.032 |  | 0.496 | 0.034 |
| PGI/II | 0.595 | 0.027 |  | 0.975 | -0.002 |
| G17 | 0.320 | -0.050 |  | 0.531 | 0.031 |
| HP +/- |  |  |  | 0.870 | -0.008 |
| GC |  |  |  |  |  |
| PGI | 0.088 | -0.078 |  | 0.292 | 0.048 |
| PGII | 0.833 | 0.010 |  | 0.857 | -0.008 |
| PGI/II | 0.355 | -0.042 |  | 0.684 | 0.019 |
| G17 | 0.141 | -0.065 |  | 0.314 | 0.044 |
| HP +/- |  |  |  | 0.865 | -0.007 |
| Note: ^a^ *P*-values to analysis EBV status and PGI, PGII, PGI/II and *H.pylori* infection status in the group of all the cases and different gastric disease groups which adjusted by ages and gender. GC, gastric cancer; AG, atrophic gastritis; CON, control group, normal stomach mucosae. | | | | | |
